# Enhancing Statistical Inference in Psychological Research via Prospective and Retrospective Design Analysis

**DOI:** 10.3389/fpsyg.2019.02893

**Published:** 2020-01-14

**Authors:** Gianmarco Altoè, Giulia Bertoldo, Claudio Zandonella Callegher, Enrico Toffalini, Antonio Calcagnì, Livio Finos, Massimiliano Pastore

**Affiliations:** ^1^Department of Developmental Psychology and Socialisation, University of Padova, Padova, Italy; ^2^Department of General Psychology, University of Padova, Padova, Italy

**Keywords:** prospective and retrospective design analysis, Type *M* and Type *S* errors, effect size, power, psychological research, statistical inference, statistical reasoning, R functions

## Abstract

In the past two decades, psychological science has experienced an unprecedented replicability crisis, which has uncovered several issues. Among others, the use and misuse of statistical inference plays a key role in this crisis. Indeed, statistical inference is too often viewed as an isolated procedure limited to the analysis of data that have already been collected. Instead, statistical reasoning is necessary both at the planning stage and when interpreting the results of a research project. Based on these considerations, we build on and further develop an idea proposed by Gelman and Carlin (2014) termed “prospective and retrospective design analysis.” Rather than focusing only on the statistical significance of a result and on the classical control of type I and type II errors, a comprehensive design analysis involves reasoning about what can be considered a plausible effect size. Furthermore, it introduces two relevant inferential risks: the exaggeration ratio or Type *M* error (i.e., the predictable average overestimation of an effect that emerges as statistically significant) and the sign error or Type *S* error (i.e., the risk that a statistically significant effect is estimated in the wrong direction). Another important aspect of design analysis is that it can be usefully carried out both in the planning phase of a study and for the evaluation of studies that have already been conducted, thus increasing researchers' awareness during all phases of a research project. To illustrate the benefits of a design analysis to the widest possible audience, we use a familiar example in psychology where the researcher is interested in analyzing the differences between two independent groups considering Cohen's *d* as an effect size measure. We examine the case in which the plausible effect size is formalized as a single value, and we propose a method in which uncertainty concerning the magnitude of the effect is formalized via probability distributions. Through several examples and an application to a real case study, we show that, even though a design analysis requires significant effort, it has the potential to contribute to planning more robust and replicable studies. Finally, future developments in the Bayesian framework are discussed.

“*If statisticians agree on one thing, it is that scientific inference should not be made mechanically*.”

Gigerenzer and Marewski (2015, p. 422)

“***A****ccept uncertainty. Be* ***t****houghtful*, ***o****pen, and* ***m****odest. Remember ‘ATOM’*.”

Wasserstein et al. (2019, p. 2)

## 1. Introduction

In the past two decades, psychological science has experienced an unprecedented replicability crisis (Ioannidis, 2005; Pashler and Wagenmakers, 2012; Open Science Collaboration, 2015) that has uncovered a number of problematic issues, including the adoption of Questionable Research Practices (John et al., 2012) and Questionable Measurement Practices (Flake and Fried, 2019), the reliance on excessively small samples (Button et al., 2013), the misuse of statistical techniques (Pastore et al., 2019), and the consequent misleading interpretation and communication of research findings (Wasserstein et al., 2019).

Whereas some important reasons for the crisis are intrinsically related to psychology as a science (Chambers, 2019), leading to a renewed recommendation to rely on strong and well-formalized theories when planning a study, the use of statistical inference undoubtedly plays a key role. Specifically, the inferential approach most widely used in psychological research, namely Null Hypothesis Significance Testing (NHST), has been strongly criticized (Gigerenzer et al., 2004; Gelman, 2018; McShane et al., 2019). As a consequence, several alternative approaches have received increasing attention, such as the use of Bayes Factors for hypothesis testing and the use of both Frequentist and Bayesian methods to estimate the magnitude of the effect of interest with uncertainty (see Kruschke and Liddell, 2018, for a comprehensive historical review).

In the current paper, we focus on an upstream—but still neglected—issue that is unrelated to the approach chosen by the researcher, namely the need for statistical reasoning, i.e., “to reason about data, variation and chance” (Moore, 1998, p. 1253), during all phases of an empirical study. Our work was inspired by the famous statistician Ronald Fisher (1890–1962), who stated that, “To consult the statistician after an experiment is finished is often merely to ask him to conduct a post-mortem examination. He can perhaps say what the experiment died of” (Fisher, 1938, p.17). Indeed, we argue that statistical inference is too often seen as an isolated procedure that is limited to the analysis of data that have already been collected. In particular, we emphasize the non-trivial importance of making statistical considerations at the onset of a research project. Furthermore, we stress that, although Fisher has ironically defined them as a “post-mortem examination,” appropriate evaluations of published results can provide a relevant contribution to the progress of (psychological) science. The ultimate goal of this paper is to increase researchers' awareness by promoting active engagement when designing their research.

To achieve this goal, we build on and further develop an idea proposed by Gelman and Carlin (2014) called “prospective and retrospective design analysis,” which is virtually absent in current research practice. Specifically, to illustrate the benefits of design analysis to the widest possible audience, we use a familiar example in psychology where the researcher is interested in analyzing the differences between two independent groups considering Cohen's *d* (Cohen, 1988) as an effect size measure.

In brief, the term *design analysis* has been proposed by Gelman and Carlin (2014) as a broader definition of power analysis—a concept that in the statistical literature traditionally indicates the determination of an appropriate sample size, at prespecified levels of Type I and Type II errors and a “plausible effects size” (Gigerenzer et al., 2004). Indeed, a comprehensive design analysis should also explicitly consider other two inferential risks: Type *M* error and Type *S* error. Type *M* error (where *M* stands for magnitude) is also known as exaggeration ratio and indicates how much a statistically significant effect is, on average, overestimated in comparison to a “plausible effect size.” Type *S* error (where *S* stands for sign) indicates the risk that a statistically significant effect is estimated in the wrong direction. These two errors will be further discussed in the subsequent paragraphs with several examples. Notably, the estimation of these errors will require an effort from psychologists to introduce their expert knowledge and hypothesize what could be considered a “plausible effect size.” As we will see later, a key aspect of design analysis is that it can be usefully carried out both in the planning phase of a study (i.e., prospective design analysis) and for the evaluation of studies that have already been conducted (i.e., retrospective design analysis).

Although the idea of a design analysis could be developed within different inferential statistical approaches (e.g., Frequentist and Bayesian), in this paper we will rely on the Neyman-Pearson (N-P) approach (Pearson and Neyman, 1928) as opposed to the widely used NHST. The rationale for this choice is that, in addition to other strengths, the N-P approach includes formalization of the *Null Hypothesis* (i.e., the absence of an effect) like NHST, but it also includes an explicit formalization of the *Alternative Hypothesis* (i.e., the magnitude of the expected effect). For a more comprehensive description of the difference between N-P and NHST approaches, we refer the reader to Gigerenzer et al. (2004).

In the next paragraphs, we will briefly review the main consequences of underpowered studies, discuss two relevant misconceptions concerning the interpretation of statistically significant results, and present a theoretical framework for design analysis, including some clarifications regarding the concept of “plausible effect size.” In section 2, through familiar examples within psychological research, the benefits of prospective and retrospective design analysis will be highlighted. In section 3, we will propose a specific method that, by explicitly taking uncertainty issues into account, could further assist researchers in evaluating scientific findings. Subsequently, in section 4, a real case study will be presented and analyzed. Finally, in section 5, we will summarize the potentials, further developments, and limitations of our proposal.

To increase readability and ensure transparency of our work, we also include two Appendices as Supplementary Material:

- **Appendix A**. A detailed description concerning the computation and the interpretation of Cohen's *d*.
- **Appendix B**. A brief explanation of the *ad-hoc* R (R Core Team, 2018) functions used in the paper. Details on how to reproduce the presented examples and on how to use our R functions for other purposes are also provided. Furthermore, the source code of our functions, PRDA.R, is freely available at the Open Science Framework (OSF) at the link https://osf.io/j8gsf/files/.

### 1.1. The Consequences of Underpowered Studies in Psychology

In 1962, Cohen called attention to a problem affecting psychological research that is still very much alive today (Cohen, 1962). Researchers seemed to ignore the statistical power of their studies—which is not considered in NHST (Gigerenzer et al., 2004)—with severe consequences for the robustness of their research findings. In the N-P approach, the power of a statistical test is defined as the probability that the test has to reject the Null Hypothesis (*H*_0_) when the Alternative Hypothesis (*H*_1_) is true. One of the problems with underpowered studies is that the probability of finding an effect, if it actually exists, is low. More importantly, if a statistically significant result (i.e., “in general,” when the observed *p*-value is <0.05 and consequently *H*_0_ is rejected; see Wasserstein et al., 2019) is obtained in an underpowered study, the effect size associated with the observed *p*-value might be “too big to be true” (Button et al., 2013; Gelman and Carlin, 2014).

This inflation of the effect sizes can be seen when examining results of replication projects, which are usually planned to have higher power than the original studies. For example, the Open Science Collaboration (2015, pp. 4–5) reported that “Overall, original study effect sizes (*M* = 0.403, *SD* = 0.188) were reliably larger than replication effect sizes (*M* = 0.197, *SD* = 0.257),” and in the Social Science Replication Project (Camerer et al., 2018, p. 637), “the effect size of the replication was on average about 50% of the original effect size.” These considerations contributed to the introduction in the literature of the term “decline effect,” defined as “the notion that science routinely observes effect sizes decrease over repeated replications for reasons that are still not well-understood” (Schooler, 2014, p. 579).

Given that underpowered studies are widespread in psychology (Cohen, 1962; Sedlmeier and Gigerenzer, 1989; Maxwell, 2004), the shrinkage of effect sizes in replications could be partially explained by the fallacy of “what does not kill statistical significance makes it stronger” (Loken and Gelman, 2017) and by the trap of the “winner's curse” (Button et al., 2013).

### 1.2. The “What Does Not Kill Statistical Significance Makes It Stronger” Fallacy and the “Winner's Curse” Trap

When a statistically significant result is obtained in an underpowered study (e.g., power = 40%), in spite of the low probability of this event happening, the result might be seen as even more remarkable. In fact, the researcher might think, “If obtaining a statistically significant result is such a rare event, and in my experiment I obtained a statistically significant result, it must be a strong one.” This is called the “what does not kill statistical significance makes it stronger” fallacy (Loken and Gelman, 2017). The reason why this is a fallacy lies in the fact that it is possible to obtain statistical significance due to the presence of many other factors that are different from the presence of a real effect. The researcher degrees of freedom, large measurement errors, and small sample sizes all contribute to the creation of noise in the data, thus inflating the perhaps true but small underlying effect. Then, if the procedure used to analyze those data is only focused on a threshold (like in NHST, with a conventional significance level of 0.05), the noise in the data allows it to pass this threshold.

**Figure 1:**
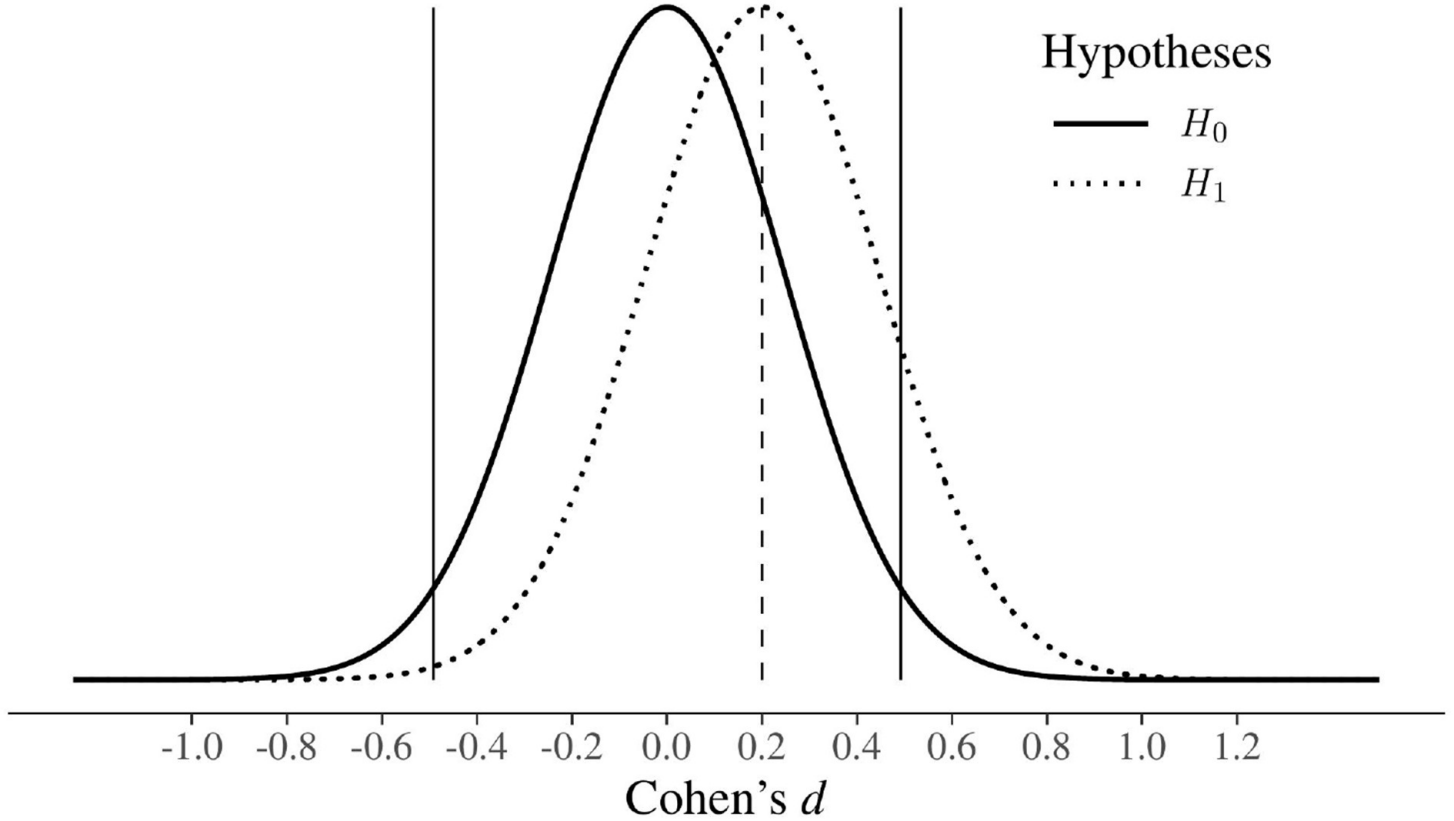
The Winner's Curse. Hypothetical study where the plausible true effect size is small (Cohen's *d* = 0.20) and a two-tailed independent samples *t*-test is performed with 33 people per group. In order to reject *H*_0_, the researcher has to overestimate the underlying true effect, which is indicated by the dashed vertical line. Note: the rejection regions of *H*_0_, given a significance level of 0.05, lie outside the vertical black lines.

In these situations, the apparent win in terms of obtaining a statistically significant result is actually a loss; “the lucky” scientist who makes a discovery is cursed by finding an inflated estimate of that effect (Button et al., 2013). This is called the “Winner's curse,” and Figure 1 shows an example of this. In this hypothetical situation, the researcher is interested in studying an effect that can plausibly be of small dimensions, e.g., Cohen's *d* of 0.20 (see Appendix A, for a detailed description of the calculation and interpretation of Cohen's *d*). If they decide to compare two groups on the outcome variable of interest, using 33 participants per group (and performing a two-tailed test), they will never be able to simultaneously reject *H*_0_ and find an effect close to what it is plausible in that research field (i.e., 0.20). In fact, in this underpowered study (i.e., based on a *d* of 0.20, the actual power is only 13%) all the effects falling in the “rejection regions” are higher than 0.49 or smaller than −0.49, and 0.20 falls in the region where the decision rules state that you cannot reject *H*_0_ under the NHST approach, and that you can accept *H*_0_ under the N-P approach.

### 1.3. Beyond Power: The Design Analysis

As we saw in the previous example, relying solely on the statistical significance of a result can lead to completely misleading conclusions. Indeed, researchers should take into account other relevant information, such as the hypothesized “plausible effect size” and the consequent power of the study. Furthermore, to assist researchers with evaluating the results of a study in a more comprehensive way, Gelman and Carlin (2014) suggested that two other relevant types of errors should be considered in addition to the traditional Type I and Type II errors, namely Type *M* and Type *S* errors (see also Gelman and Tuerlinckx, 2000; Lu et al., 2019). Specifically, a Type *M* [magnitude] error or *exageration ratio* can be viewed as the expected average overestimation of an effect that emerges as statistically significant, whereas a Type *S* [sign] error can be viewed as the probability of obtaining a statistically significant result in the opposite direction with respect to the sign of the hypothesized plausible effect size.

Based on this consideration, Gelman and Carlin (2014) proposed the term “*design analysis*” to broadly identify the analysis of the properties of different studies, such as their statistical power as well as Type *M* and Type *S* errors. Moreover, as is shown in the next paragraph, in design analysis particular emphasis is given to the elicitation and formalization of what can be considered a *plausible effect size* (see also paragraph 1.4) for the study of interest. In this regard, it is important to make a clarification. Although Gelman and Carlin (2014) developed a design analysis relying on an unstandardized effect size measure (i.e., the difference between two means), we have, in this paper, adapted their method to deal with Cohen's *d*, a standardized measure of effect size that is more commonly used in psychology (see Appendix A for more details on the reasons that motivated this choice).

Given these premises, the steps to perform design analysis using Cohen's *d* as a measure of effect size can be summarized in three steps:

1. A plausible effect size for the study of interest needs to be identified. Rather than focusing on data at hand or on noisy estimates of a single pilot study, the formalization of a plausible effect size should be based on an extensive theoretical literature review and/or on meta-analyses. Moreover, specific tools (see for example Zondervan-Zwijnenburg et al., 2017; O'Hagan, 2019; Zandonella Callegher et al., 2019) that allow for the incorporation of expert knowledge can also be considered to increase the validity of the plausible effect size elicitation process^1^.
2. Based on the experimental design of the study of interest (in our case, a comparison between two independent groups), a large number of simulations (i.e., 100,000) will be performed according to the identified plausible effect size. This procedure serves to provide information about what to expect if the experiment is replicated an infinite number of times and assuming that the pre-identified plausible effect is true.
3. Given a fixed level of Type I error (e.g., 0.05), power as well as type *M* and type *S* errors will be calculated. Specifically, power will be estimated as the ratio between the number of significant results obtained and the number of replicates (i.e., the higher the power, the higher the probability of detecting the plausible effect). A Type *M* error will be estimated as the ratio between the mean of the absolute values of the statistically significant replicated effect sizes and the plausible effect size. In this case, larger values indicate an expected large overestimation of the plausible effect size. Type *S* error will be the ratio between the number of significant results with opposite signs with regard to the plausible effect size and the total number of significant results. Put in other terms, a type *S* error estimates the probability of obtaining a significant result in the wrong direction.

Although the procedure may seem complex to implement, we have here https://osf.io/j8gsf/files/ (see also Appendix B) made available some easy-to-use R functions that allow others to perform different types of design analysis, even for less experienced users. The same functions will also be used in the examples and application presented in this paper.

To get a first idea of the benefits of design analysis, let us re-analyze the hypothetical study presented in Figure 1. Specifically, given a plausible effect size equal to *d* = 0.20 and a sample size of 33 participants per group, a design analysis will highlight the following information: power = 13%, Type *M* error = 3.11, and Type *S* error = 2%. Despite the low power, which shows that the study has only a 13% probability of detecting the plausible effect size, a type *M* error explicitly indicates that the expected overestimate of a result that will emerge as statistically significant is around three times the plausible effect. Furthermore, given a Type *S* error of 2%, there is also a non-negligible probability of obtaining a significant result in the wrong direction. Overall, the results of design analysis clearly tell the researcher that the study of interest could provide very poor support to both the existence and non-existence of a plausible effect size.

Another advantage of design analysis, which will be better explored in the following sections, is that it can be effectively used in the planning phase of a study, i.e., *prospective design analysis*, as well as in the evaluation of already obtained study results, i.e., *retrospective design analysis*. For example, in prospective design analysis, considerations concerning power as well as Type *M* and Type *S* errors could assist researchers in deciding the appropriate sample size for detecting the effect of interest (if it actually exists). In a retrospective design analysis, power as well as Type *M* and Type *S* errors (always calculated using the theoretically plausible effect size) can be used to obtain information about the extent to which the results of the study could be exaggerated and/or in the wrong direction. Most importantly, we believe that, engaging in a retrospective design analysis helps researchers to recognize the role of uncertainty and to make more reasonable statistical claims, especially in those cases at risk of falling in the aformentioned “Winner's Curse” trap.

**Figure 2:**
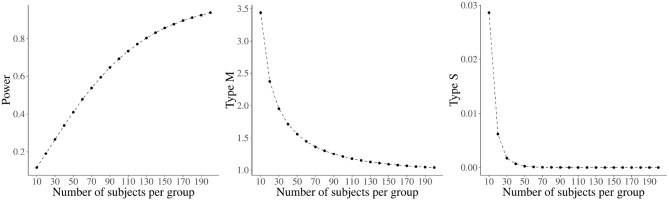
Relationship between sample size and Power, Type *M*, and Type *S* for a Cohen's *d* of 0.35 in an independent samples *t*-test. Type I error is set at 0.05.

In conclusion, it is important to note that whatever the type of design analysis chosen (prospective or retrospective), the relationships between power, type *M* error, and type *S* error are the same. For illustrative purposes, these relationships are graphically displayed as a function of sample size in Figure 2. A medium-to-small effect of *d* = 0.35 (i.e., a reasonable average effect size for a psychological study in the absence of other relevant information, see also section 4) was considered as a plausible effect size, and Type I error was set at 0.05.

As expected, power increases as sample size increases. Moreover, type *M* and type *S* errors decrease as the size of the sample increases, with the latter showing a much steeper decrease.

From an applied perspective, issues with type *M* and *S* errors emerge with underpowered studies, which are very common in psychological research. Indeed, as can be seen in Figure 2, for a power of 40% (obtained with 48 participants per group), the type *M* error reaches the worrisome value of 1.58; for a power around 10% (i.e., with 10 participants per group), even a type *S* error becomes relevant (around 3%).

### 1.4. What Does “Plausible Effect Size” Mean?

“*Thinking hard about effect sizes is important for any school of statistical inference [i.e., Frequentist or Bayesian], but sadly a process often neglected.”*

Dienes (2008, p. 92)

The main and most difficult point rests on deciding what could be considered a “plausible effect size.” Although this might seem complex, studies are usually not developed in a void. Hypotheses are derived from theories that, if appropriately formalized in statistical terms, will increase the validity of the inferential process. Furthermore, researchers are commonly interested in knowing the size and direction of effects; as shown above, this corresponds to control for a Type *M* [magnitude] error and a type *S* [sign] error.

From an epistemological perspective, Kruschke (2013) suggests an interesting distinction between *strong theories* and *weak theories*. Strong theories are those that try to make precise predictions and could be, in principle, more easily disconfirmed. For example, a strong theory could hypothesize a medium-sized positive correlation between two variables. In contrast, weak theories make broader predictions, such as the hypothesis that two variables are correlated without specifying the strength and direction of the correlation (Dienes, 2008). The former type allows many more research findings to disconfirm the hypothesis, whereas the latter type allows only the result of no correlation to disconfirm it. Specifically, following Karl Popper (1902–1994), it could be argued that theories explaining virtually everything and that are hard to disconfirm risk being out of the realm of science. Thus, scientific theories should provide at least a hint regarding the effect that is expected to be observed.

A challenging point is to establish the dimension of this effect. It might seem paradoxical that the researcher must provide an estimate of the effect size before running the experiment given that they will conduct the study with the precise aim of finding what that estimate is. However, strong theories should allow to make such predictions, and the way in which science accumulates should provide increasing precision to these predictions.

In practice, it might be undesirable to simply take the estimate found in a pilot study or from a single previous study published in the literature as the “plausible effect size.” In fact, the plausible effect size refers to what could be approximately the true value of the parameter in the population, whereas the results of pilots or single studies (especially if underpowered) are noisy estimates of that parameter.

In line with Gelman and Carlin (2014), we suggest the use of information outside the data at hand, such as literature reviews and/or meta-analyses taking into account issues concerning publication bias (Borenstein et al., 2009). Moreover, as stated in the previous paragraph, promising procedures to elicit and formalize expert knowledge should also be considered. It is important to note that, whatever the procedures, all assumptions that will lead to the identification of a plausible effect size must be communicated in a transparent manner, thus increasing the information provided by a study and ensuring more reasonable statistical claims related to the obtained results, regardless of whether they are significant or not.

As we have seen, the identification of a plausible effect size (or a series of plausible effect sizes to explore different scenarios) requires significant effort from the researcher. Indeed, we believe that this kind of reasoning can make a substantial contribution to the planning of robust and replicable studies as well as to the efficient evaluation of obtained research findings.

To conclude, we leave the reader with a question: “All other conditions being equal, if you had to evaluate two studies of the same phenomenon, the first based on a formalization of the expected plausible effect sizes of interest that is as accurate as possible, and the second one in which the size of the effects of interest was not taken into account, the findings of which study would you believe the most?” (R. van de Schoot, personal communication).

## 2. Prospective and Retrospective Design Analysis

To highlight the benefits of design analysis and to make familiar the concepts of Type *M* and Type *S* errors, we will start with a simple example that is well-known in psychological research, i.e., the comparison between the means of two independent groups^2^.

In particular, the goal of our hypothetical case study was to evaluate the differences between two treatments that aim to improve a cognitive ability called *Y*. Both treatments have the same cost, but the first is innovative, whereas the second is traditional. To this end, the researchers recruited a sample of participants who were homogeneous with respect to pre-specified relevant study variables (i.e., age, IQ, etc.). Next, they randomly assigned each participant to one of the two conditions (i.e., innovative vs. traditional treatment). After the treatment phase was completed, the means of the two groups were compared.

### 2.1. Prospective Design Analysis

Before collecting data, the researchers planned the appropriate sample size to test their hypotheses, namely that there was a difference between the means of *G*1 (the group to which the innovative treatment was administered) and *G*2 (the group to which the traditional treatment was administered) vs. there was no difference.

After an extensive literature review concerning studies theoretically comparable to their own, the researchers decided that a first reasonable effect size for the difference between the innovative and the traditional treatment could be considered equal to a Cohen's *d* of 0.30 (see Appendix A for a detailed description of the calculation and interpretation of Cohen's *d*). Due to the possible presence of publication bias (Borenstein et al., 2009), which could lead to an overestimation of the effects of published studies, the researchers decided to be more conservative about the estimate of their plausible effect size. Thus, they decided to consider a Cohen's *d* of 0.25. Eventually, all researchers agreed that a Cohen's *d* of 0.25 could also represent a clinically relevant effect in order to support the greater efficacy of the innovative treatment.

Based on the above considerations, the researchers started to plan the sample size for their study. First, they fixed the Type I error at 0.05 and—based on commonly accepted suggestions from the psychological literature—fixed the power at 0.80. Furthermore, to explicitly evaluate the inferential risks connected to their choices, they calculated the associated Type *M* and Type *S* errors.

Using our R function design_analysis, they obtained the following results:


  > design_analysis (d=0.25, power=0.80)
       d  power      n   typeS typeM
    0.25   0.80 252.00    0.00  1.13


Based on the results, to achieve a power of 0.80, a sample size of 252 for each group was needed (i.e., total sample size = 504). With this sample size, the risk of obtaining a statistically significant result in the wrong direction (Type *S* error) was practically 0, and the expected exaggeration ratio (Type *M* error) was 1.13. In other words, the expected overestimation related to effects that would emerge as statistically significant would be around 13% of the hypothesized plausible effect size.

Although satisfied in terms of expected type *S* and type *M* risks, the researchers were concerned about the economic feasibility of recruiting such a “large” number of subjects. After a long discussion, they decided to explore which inferential risks would result for a lower level of power, namely 60%^3^.

Using the function design_analysis


  > design_analysis (d=0.25, power=0.60)
       d  power      n   typeS typeM
    0.25   0.60 158.00    0.00  1.30


they discovered that: (1) the overall required sample size was considerably smaller (from 504 to 316 = 158 × 2), thus increasing the economic feasibility of the study; (2) the Type *S* error remained negligible (0%); and (3) the exaggeration ratio considerably increased (from 1.13 to 1.30); thus, an effect that will emerge as statistically significant will be on average 130% of the hypothesized plausible effect size.

The researchers had to make a decision. From a merely statistical point of view, the optimal choice would be to consider a power of 80% that is associated with a Type *M* error of 1.13 (i.e., mean overestimation of ~10%) and a negligible Type *S* error close to zero. However, it is important to highlight that these values cannot be considered universal benchmarks. Indeed, other relevant aspects must be considered, such as the practical implications of an expected overestimation of the plausible effect size, the phase of the study (i.e., preliminary/exploratory, intermediate, or final/confirmatory), and feasibility constraints.

Whatever the decision, the researchers must be aware of the inferential risks related to their choice. Moreover, when presenting the results, they must be transparent and clear in communicating such risks, thus highlighting the uncertainty associated with their conclusions.

### 2.2. Retrospective Design Analysis

To illustrate the usefulness of retrospective design analysis, we refer to the example presented in the previous paragraph. However, we introduce three new scenarios that can be considered as representative of what commonly occurs during the research process:

- **Scenario 1 (S1): Evaluating sample size based on a single published study**^4^
  Imagine that the researchers decide to plan their sample size based on a single published study in the phase of formalizing a plausible effect size, either because the published study presents relevant similarities with their own study or because there are no other published studies available.
  *Question*: What type of inferential risks can be associated with this decision?
  *Issues*: Using a single study as a reference point without considering other sources (e.g., theoretical framework, expert opinion, or a meta-analysis), especially when the study has a low sample size and/or the effect of interest is small, can lead to use an excessively optimistic estimate of the effect size when planning an appropriate sample size (Gelman and Carlin, 2014).
- **Scenario 2 (S2): Difficulty in recruiting the planned number of research participants**
  Imagine that, due to unforeseen difficulties (e.g., insufficient funding), the researchers are not able to recruit the pre-planned number of participants as defined based on prospective design analysis.
  *Question*: How do you evaluate the inferential risks associated with the new reduced sample size? How do you communicate the obtained results?
  *Issues*: Researchers are often tempted to evaluate the results of their study based on the observed effect size. This procedure, known as “*post-hoc* power analysis,” has been strongly criticized, and many statistical papers explicity advise against its use (see for example, Goodman and Berlin, 1994; Gelman, 2019). Indeed, to evaluate the information provided by the obtained results, researchers should use the *a priori* plausible effect size, i.e., the one formalized before collecting their data.
- **Scenario 3 (S3): No prospective design analysis because the number of participants is constrained**
  Imagine the number of participants involved in the study have specific characteristics that make it impossible to yield a large sample size, or that the type of treatment is particularly expensive and cannot therefore be tested on a large sample. In this case, the only possibility is to recruit the largest possible number of participants.
  *Question*: What level of scientific quality can be provided by the results?
  *Issues*: Although study results can provide a useful contribution to the field, there are several associated inferential risks that the researchers need to communicate in a transparent and constructive way.

As we will see below, retrospective design analysis can be a useful tool to deal with the questions and the issues raised across all three scenarios.

For the sake of simplicity and without loss of generalizability, suppose that in each of the three scenarios the researchers obtained the same results (see Table 1).

**Table 1:** Comparison of the cognitive skill *Y* between the two groups.

**Group**	***n***	***M***	***SD***	***t* (df)**	***p***	**Cohen's *d* (95% CI)**
Innovative treatment	31	114	16	3.496 (60)	0.001	0.90 (0.38–1.43)
Traditional treatment	31	100	15			

At a first glance, the results indicated a statistically significant difference in favor of the innovative treatment (see Table 1), with a large effect size (i.e., *d* = 0.90). However, the 95% confidence interval for Cohen's *d* was extremely wide, suggesting that both medium-small (i.e., *d* = 0.38) and very large (i.e., *d* = 1.43) effects were consistent with the observed data.

A closer look indicated that the estimated effect size seemed too large when compared with the initial guess of the researchers (i.e., *d* = 0.25). Furthermore, an estimated *d* of 0.90 seemed, in general, implausibly large for a difference between two cognitive treatments (see also Appendix A). The latter interpretation seemed to be also supported by the fact that the hypothesized plausible effect size was not even included in the estimated confidence interval. Overall, in order to prevent the aforementioned “Winner's Curse” and “What Does Not Kill Statistical Significance Makes It Stronger” heuristics, results had to be evaluated and eventually communicated with caution and skepticism.

To obtain a clearer picture of the inferential risks associated with the observed results, we performed a retrospective design analysis using *d* = 0.25 as plausible effect size and 31 participants per group as sample size:


  > design_analysis (n=31, d=0.25)
  power typeS typeM
   0.16  0.01  2.59


As can be seen, the power was markedly low (i.e., only 16%) and the Type *M* error even suggested an expected overestimation around two and a half times the plausible effect size. Lastly, the Type *S* error, although small, indicated a 1% risk of obtaining a significant result in the wrong direction (i.e., the traditional treatment is better than the innovative treatment). Let's see how this information could be helpful to deal with the three presented scenarios.

In S1, the researchers took a single noisy estimate as the plausible effect size from a study that found a “big” effect size (e.g., 0.90). The retrospective design analysis showed what happens if the plausible effect size is, in reality, much smaller (i.e., 0.25). Specifically, given the low power and the high level of Type *M* error, researchers should abandon the idea of planning their sample size based on a single published study. Furthermore, issues regarding the presence of Questionable Research Practices (John et al., 2012; Arrison, 2014) and Questionable Measurement Practices (Flake and Fried, 2019) in the considered published study must at least be explored. From an applied perspective, researchers should continue with a more comprehensive literature review and/or consider the opportunity of using an effect size elicitation procedure that is based on expert knowledge (Zondervan-Zwijnenburg et al., 2017; O'Hagan, 2019).

In S2, to check the robustness of their results, researchers might initially be tempted to conduct a power analysis based on their observed effect size (*d* = 0.90). Acting in this way, they would obtain a completely misleading *post-hoc* power of 94%. In contrast, the results of the retrospective design analysis based on the a-priori plausible effect size (*d* = 0.25) highlight the high level of inferential risks related to the observed results. From an applied perspective, researchers should be very skeptical about their observed results. A first option could be to replicate the study on an independent sample, perhaps asking for help from other colleagues in the field. In this case, the effort to recruit a larger sample could be well-justified based on the retrospective design analysis.

In S3, given the low power and the high level of Type *M* error, results should be presented as merely descriptive by clearly explaining the uncertainty that characterizes them. Researchers should first reflect on the possibility of introducing improvements to the study protocol (i.e., improving the reliability of the study variables). As a last option, if improvements are not considered feasible, the researchers might consider not continuing their study.

Despite its advantages, we need to emphasize that design analysis should not be used as an *automatic problem solver machine*: “Let's pull out an effect size …let me see the correct sample size for my experiment.” In other words, to obtain reliable scientific conclusions there is no “free lunch.” Rather, psychologists and statisticians have to work together, case by case, to obtain a reasonable effect size formalization and to evaluate the associated inferential risks. Furthermore, researchers are encouraged to explore different scenarios via a sensitivity analysis (see section 4) to better justify and optimize their choices.

## 3. Incorporating Uncertainty Concerning Effect Size Formalization in Retrospective Design Analysis

As shown in the previous examples, a key point both in planning (i.e., prospective design analysis) and in evaluating (i.e., retrospective design analysis) a study is the formalization of a plausible effect size. Using a single value to summarize all external information and previous knowledge with respect to the study of interest can be considered an excessive simplification. Indeed, all uncertainty concerning the magnitude of the plausible effect size is not explicitly taken into consideration. In particular, the level of heterogeneity emerging from the examination of published results and/or from different opinions of the consulted experts, which can be poorly formalized. The aim of this paragraph is to propose a method that can assist researchers with dealing with these relevant issues. Specifically, we will focus on the evaluation of the results of a study (i.e., retroprospective design analysis).

Our method can be summarized in the three steps: (1) defining a lower and an upper bound within which the plausible effect size can reasonably vary; (2) formalizing an appropriate probability distribution that reflects how the effect size is expected to vary; and (3) conducting the associated analysis of power, Type *M* error, and Type *S* error.

To illustrate the procedure, we use the study presented in Table 1 as a reference. Let us now hypothesize that, after a thorough evaluation of external sources, the researchers conclude that a plausible effect size could reasonably vary between 0.20 and 0.60 (instead of specifying a too simplistic single-point value). It should be noted that, from a methodological perspective, the specification of a “plausible interval” can be considered an efficient and informative starting point to elicit the researchers' beliefs (O'Hagan, 2019).

**Figure 3:**
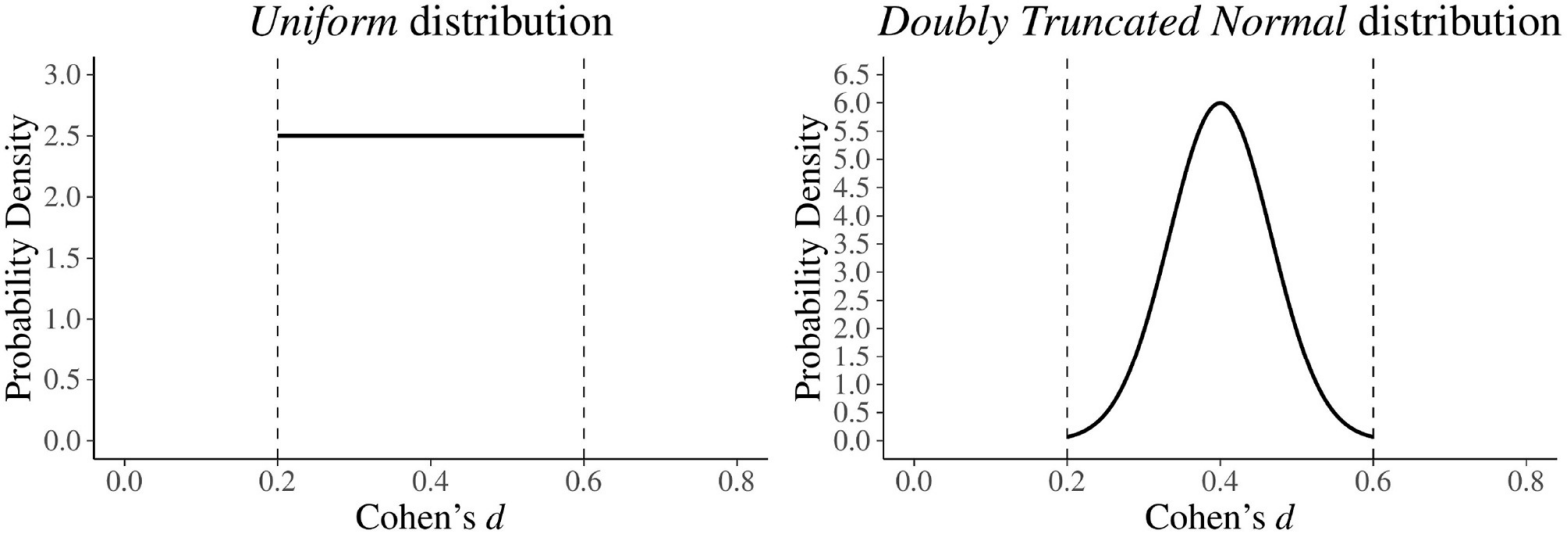
Different ways to formalize a plausible interval for the effect size *d*. In the left panel, a Uniform distribution with lower bound = 0.20 and upper bound = 0.60 is used. In the right panel, a doubly truncated Normal distribution with lower bound = 0.20, upper bound = 0.60, mean = 0.40, and standard deviation equal to $\frac{1}{6}$ the length of the interval (i.e., $\frac{0.60-0.20}{6}=0.067$) is used.

At this point, a first option could be to assume that, within the specified interval, all effect size values have the same probability of being true. This assumption can be easily formalized using a Uniform distribution, such as the one shown in Figure 3 (left panel).

However, from an applied point of view it is rare for the researcher to expect that all values within the specified interval have the same plausibility. Indeed, in general conditions, it is more reasonable to believe that values around the center of the interval (i.e., 0.40 in our case) are more plausible, and that their plausibility gradually decreases as they move away from the center. This expectation can be directly formalized in statistical terms using the so-called “doubly truncated Normal distribution.” On an intuitive level (for a more complete description see Burkardt, 2014), the doubly truncated Normal distribution can be seen as a Normal distribution whose values are forced to vary within a specific closed interval. In case of the formalization of the plausible effect size, we propose the use of doubly truncated Normal distribution with several parameters: a lower and an upper bound according to the pre-specified plausible interval, a mean fixed at the center of the interval, and a standard deviation that reflects the hypothesized uncertainty around the center. A standard deviation of $\frac{1}{10}$ the length of the chosen interval will produce a substantially Normal distribution. Higher values, like $\frac{1}{6}$ the length of the interval (see right panel of Figure 3) will lead to normal-like distributions with increased probability on the tails, thus reflecting greater uncertainty around the center.

Coming back to our example, suppose that the researchers want to evaluate the study of interest assuming a plausible interval for Cohen's *d* as the one represented in Figure 3. Using the *ad-hoc* function design_est^5^ they will obtain this information :


  > design_est(n1=31, n2=31, target_d_limits=
  c(0.20,0.60), distribution="normal")
  power typeS typeM
   0.35  0.00  1.73


To summarize, this information suggests that the results of the study of interest (see Table 1) should be taken very cautiously. Indeed, the expected power was low (35%), and the expected overestimation of the most plausible effect size (i.e., *d* = 0.40) was around 73%. Furthermore, it is important to note that the observed effect size of 0.90 fell abundantly outside the pre-specified plausible interval of 0.20–0.60, thus supporting the idea that the study of interest clearly overestimated the actual magnitude of the effect.

In general, when the observed effect size falls outside the pre-specified plausible interval, we can conclude that the observed study is not coherent with our theoretical expectations. On the other hand, we could also consider that our plausible interval may be unrealistic and/or poorly formalized. In these situations, researchers should be transparent and propose possible explanations that could be very helpful to the understanding of the phenomenon under study. Although this way of reasoning requires a notable effort, the information provided will lead to a more comprehensive inference than the one deriving from a simplistic dichotomous decision (i.e., “reject / do not reject”) typical of the NHST approach. Indeed, in this approach the hypotheses are poorly formalized, and power, Type *M* error, and Type *S* error are not even considered.

## 4. An Illustrative Application to a Case Study

To illustrate how design analysis could enhance inference in psychological research, we have considered a real case study. Specifically, we focused on Study 2 of the published paper “A functional basis for structure-seeking: Exposure to structure promotes willingness to engage in motivated action” (Kay et al., 2014).

The paper presented five studies arising from findings showing that human beings have a natural tendency to perceive structure in the surrounding world. Various social psychology theories propose plausible explanations that share a similar assumption that had never been tested before: that perceiving a structured world could increase people's willingness to make efforts and sacrifices toward their own goals. In Study 2, the authors decided to test this hypothesis by randomly assigning participants to two different conditions differing in the type of text they had to read. In the “random” condition, the text conveyed the idea that natural phenomena are unpredictable and random, whereas in the “structure” condition the phenomena were described as predictable and systematic. The outcome measure was the willingness to work toward a goal that each participant chose as their “most important.” The expected result was that participants in the “structure” condition would report a higher score in the measure of goal-directed behavior than those in the “random” condition.

### 4.1. Prospective Design Analysis

As we saw in the previous paragraphs, before collecting data it is fundamental to plan an appropriate sample size via prospective design analysis. In this case, given the relative novelty of Study 2, was hard to identify a single plausible value for the size of the effect of interest. Rather, it seemed more reasonable to explore different scenarios according to different plausible effect sizes and power levels. We started with a minimum *d* of 0.20, so that the study was planned to detect at least a “small” effect size. If the final results did not reach statistical significance, the researchers could conclude that it was unlikely that the true effect was equal to or >0.20, and they could eventually decide whether it would be worth it to replicate the study, perhaps by modifying their protocol.

As the most plausible effect size, we considered *d* = 0.35, which could be considered—at least in our opinion—a typical average level with which to test a hypothesis in psychological research in the absence of informative external sources (see for example the results reported in Open Science Collaboration, 2015)^6^. As extrema ratio, we included also a *d* of 0.5, which, in the words of Jacob Cohen, can be referred to as “differences that are large enough to be visible to the naked eye” (see Cohen 1988, p. 26 and Appendix A), and that, given the experiment under investigation, could be viewed as an extremely optimistic guess. Finally, to take issues concerning the feasibility of the study into account, we also considered two levels of power, namely 80 and 60%.

Overall, our “sensitivity” prospective design analysis (see Table 2) suggested that the sample size chosen by the authors (*n* = 67) was inadequate. Indeed, even in the least reasonable scenario (*d* = 0.50, power = 0.60), a minimum of 80 participants is required. Furthermore, is should be noted, that the associated Type *M* error was considerably high, i.e., 130%, signaling a high risk of overestimating the plausible effect.

**Table 2:** Sample size, Type *M* and Type *S* error by power and plausible effect size. Type I error is fixed at 0.05.

**Power**	**Cohen's *d***	***n* (per sample)**	**Total *n***	**Type *M* error**	**Type *S* error**
0.80	0.20	392	784		
	0.35	130	260	1.13	0.00
	0.50	64	128		
0.60	0.20	244	488		
	0.35	82	164	1.30	0.00
	0.50	40	80		

A good compromise could be to consider the second scenario (*d* = 0.35, power = 0.80), which requires a total sample size of 260, guaranteeing optimal control of the Type *M* error. After conducting the study with this sample size, a significant result would lead to the acceptance of the researcher's hypothesis, while a non-significant result would indicate that, if an effect exists, the effect would presumably be <0.35. Whatever the result, the researchers could eventually present their findings in a transparent and informative way. In any case, the results could be used to improve scientific progress. As an example, other researchers could fruitfully use the observed results as a starting point for a replication study.

### 4.2. Retrospective Design Analysis

Let us now evaluate Study 2 from a retrospective point of view. Based on their results [*M*_structure_ = 5.26, *SD*_structure_ = 0.88, *M*_random_ = 4.72, *SD*_random_ = 1.32, *n*_total_ = 67; *t*_(65)_ = 2.00, *p* = 0.05, Cohen's*d* = 0.50]^7^, the authors concluded that “participants in the structure condition reported higher willingness to expend effort and make sacrifices to pursue their goal compared to participants in the random condition.” Kay et al. (2014, p. 487), thus supporting their initial hypothesis.

To evaluate the inferential risks associated with this conclusion, we ran a sensitivity retrospective design analysis on the pre-identified plausible effect sizes (i.e., *d* = 0.20, *d* = 0.35, *d* = 0.50).

In line with the results that emerged from the prospective analysis, the retrospective design analysis indicated that the sample size used in Study 2 exhibited high inferential risks. In fact, both for a plausible effect of *d* = 0.20 (power = 0.13, type *M* = 3.06, type *S* = 2%) and for a plausible effect of *d* = 0.35 (power = 0.29, type *M* = 1.86, type *S* = 0%), the power was very low, and the Type *M* error reached worrying levels. For a *d* of 0.50 (chosen on the basis of plausible effects and not based on the results observed in Study 2), the Type *M* error was 1.40, indicating an expected overestimate of 40%. Furthermore, the power was 0.52, suggesting that if we replicated the study on a new sample with the same number of participants, the probability of obtaining a significant result would be around the chance level.

We also evaluated the results of Study 2 by performing a retrospective design analysis using the method presented in section 3. Specifically, we used a doubly truncated normal distribution centered at 0.35 (i.e., the most plausible effect size) with a plausible interval of 0.20–0.50. As could be expected, the results (i.e., power = 0.29, type *M* = 1.86, type *S* = 0%) substantially confirmed what emerged from the sensitivity retrospective design analysis.

In summary, our retrospective design analysis indicated that, although statistically significant, the results of Study 2 were inadequate to support the authors' conclusions.

As mentioned at the beginning of this paragraph, Study 2 by Kay et al. (2014) was selected for illustrative purposes in a constructive perspective. For a more comprehensive picture, we invite interested readers to consult the “Many Labs 2 project” (Klein et al., 2018), which showed that with a large sample size (*n* = 6506) the original conclusion of Study 2 cannot be supported (i.e., *t*(6498.63) = −0.94, *p* = 0.35, *d* = −0.02, 95*%CI* = [−0.07, 0.03], and neither can the subsequent response of the original authors (Laurin et al., 2018).

## 5. Discussion and Conclusions

In psychological research, statistical inference is often viewed as an isolated procedure that limits itself to the analysis of data that have already been collected. In this paper, we argue that statistical reasoning is necessary both at the planning stage and when interpreting the results of a research project. To illustrate this concept, we built on and further developed Gelman and Carlin's (2014) idea of “prospective and retrospective design analysis.”

In line with recent recommendations (Cumming, 2014), design analysis involves in-depth reasoning on what could be considered as a plausible effect size within the study of interest. Specifically, rather than focusing on a single pilot or published study, we underlined the importance of using information outside the data at hand, such as extensive literature reviews and meta-analytic studies, taking issues related to publication bias into account. Furthermore, we introduced the potentials of elicitation of expert knowledge procedures (see for example Zondervan-Zwijnenburg et al., 2017; O'Hagan, 2019). Even though these procedures are still under-utilized in psychology, they could provide a relevant contribution to the formalization of research hypotheses.

Moving beyond the simplistic and often misleading distinction between significant and non-significant results, a design analysis allows researchers to quantify, consider, and explicitly communicate two relevant risks associated with their inference, namely exaggeration ratio (Type *M* error) and sign error (Type *S* error). As illustrated in the paper, the evaluation of these risks is particularly relevant in studies that investigate small effect sizes in the presence of high levels of intra- and inter-individual variability, with a limited sample size—a situation that is quite common in psychological research.

Another important aspect of design analysis is that it can be usefully carried out both in the planning phase of a study (i.e., prospective design analysis) and to evaluate studies that have already been conducted (i.e., retrospective design analysis), reminding researchers that the process of statistical inference should start before data collection and does not end when the results are obtained. In addition, design analysis contributes to have a more comprehensive and informative picture of the research findings through the exploration of different scenarios and according to different plausible formalizations of the effect of interests.

To familiarize the reader with the concept of design analysis, we included several examples as well as an application to a real case study. Furthermore, in addition to the classic formalization of the effect size with a single value, we proposed an innovative method to formalize uncertainty and previous knowledge concerning the magnitude of the effect via probability distributions within a Frequentist framework. Although not directly presented in the paper, it is important to note that this method could also be efficiently used to explore different scenarios according to different plausible probability distributions.

Finally, to allow researchers to use all the illustrated methods with their own data, we also provided two easy-to-use R functions (see also Appendix B), which are available at the Open Science Framework (OSF) at the link https://osf.io/j8gsf/files/.

For the sake of simplicity, in this paper we limited our consideration to Cohen's *d* as an effect size measure within a Frequentist approach. However, the concept of design analysis could be extended to more complex cases and to other statistical approaches. For example, our R functions could be directly adapted to other effect size measures, such as Hedges' *g*, Odds Ratio, η^2^, and *R*^2^. Moreover, concerning the proposed method to formalize uncertainty and prior knowledge, other probability distributions beyond those proposed in this paper (i.e., the uniform and the doubly truncated normal) could be easily added. This was one of the main reasons behind the choice to use resampling methods to estimate power as well as Type *M* and Type *S* errors instead of using an analytical approach.

Also, it is important to note that our considerations regarding design analysis could be fruitfully extended to the increasingly used Bayesian methods. Indeed, our proposed method to formalize uncertainty via probability distributions finds its natural extension in the concept of Bayesian prior. Specifically, design analysis could be useful to evaluate the properties and highlight the inferential risks (such as Type *M* and Type *S* errors) associated with the use of Bayes Factors and parameter estimation with credible Bayesian intervals.

In summary, even though a design analysis requires significant effort, we believe that it has the potential to contribute to planning more robust studies and promoting better interpretation of research findings. More generally, design analysis and its associated way of reasoning helps researchers to keep in mind the inspiring quote presented at the beginning of this paper regarding the use of statistical inference: “Remember ATOM.”

## Data Availability Statement

All R scripts used to reproduce the examples presented in the paper are reported in the article/Supplementary Material.

## Author Contributions

GA conceived the original idea and drafted the paper. GB, CZ, and ET contributed to the development of the original idea and drafted sections of the manuscript. MP and GA wrote the R functions. GA, MP, and CZ took care of the statistical analysis and of the graphical representations. LF and AC provided the critical and useful feedback. All authors contributed to the manuscript revision, read, and approved the submitted version.

### Conflict of Interest

The authors declare that the research was conducted in the absence of any commercial or financial relationships that could be construed as a potential conflict of interest.

## Footnotes

^1^To obtain a more comprehensive picture of the inferential risks associated with their study, we suggest that researchers inspect different scenarios according to different plausible effect sizes and perform more than one design analysis (see for example our application to a real case study in section 4).

^2^We remind the reader that Appendix B provides a brief explanation of the *ad-hoc* R functions used in the paper as well as details on how to reproduce the presented examples and on how to use our R functions for other purposes. The source code of our functions, functions_PRDA.R, is available at the link https://osf.io/j8gsf/files/.

^3^Specifically, we agree with Gelman (2019) that an 80% level of power should not be used as an automatic routine, and that requirements of 80% power could encourage researchers to exaggerate their effect sizes when planning sample size.

^4^Even though, in this paper, we strongly recommend that one does not plan the sample size based on a single study, we propose this example to further emphasize the inferential risks associated with the information provided by a single underpowered study.

^5^The idea behind this function is simple. First, we sample a large number (e.g., 100,000) of effect sizes *d* from the probability distribution associated with the plausible interval. Then, for each *d* we calculate power, type *M* error, and type *S* error based on the sample size of the two groups involved in the comparison, and we consider the center of the plausible interval as the most plausible effect size. In this way, a distribution for each of the three indices is finally obtained. In the output of the function, the means of the three distributions are presented as a summary value. For additional details, see Appendix B, which also shows (in section “design_est”) how to obtain the expected distribution of power as well as Type *M* and Type *S* errors, given the plausible interval for *d*.

^6^In the Open Science Collaboration (2015), the authors conducted replications of 100 experimental and correlational studies published in three psychology journals using high-powered designs and original materials when possible. They found an average effect size of *r* = 0.197, i.e., *d* = 0.41. Given the heterogeneity of the 100 studies, we propose the use of a more conservative value to represent a typical average effect in psychology. Overall, it should be noted that all the pre-specified values of *d*, albeit plausible, are not based on a thorough theoretical revision and/or on the formalized knowledge of experts in the field. Indeed, an appropriate use of the latter two external sources would undoubtedly contribute to producing more reliable results, but a discussion of these strategies is beyond the scope of this paper.

^7^The authors reported only the total sample size (*n* = 67). Since participants were randomly assigned to each of the two experimental conditions, in the following we assumed, without loss of generalizability, that 34 participants were assigned to the “structure” condition and 33 to the “random” condition.
