# Supplementary material for: Enhancing Statistical Inference in Psychological Research via Prospective and Retrospective Design Analysis
Source: Front Psychol. 2020 Jan 14;10:2893. doi: 10.3389/fpsyg.2019.02893 (PMC6970975; doi:10.3389/fpsyg.2019.02893)
Supplement: Supplementary file 1 [file Presentation_1.pdf]

Appendices

APPENDIX A: COHEN'S D

Cohen's d definition

When considering the difference between two groups, the raw mean difference has an intuitive and meaningful interpretation if the outcomes are measured using a widespread scale (i.e., meters for height or kilos for weight). This allows us to easily share and compare the results of different studies. However, in the behavioural and social sciences, the same outcomes are often evaluated with different instruments, each of which has its own scale (i.e., questionnaire or test scores). Thus, it is difficult to interpret and compare study results when the outcomes are assessed with different measurement scales.

To overcome this issue, Cohen (1988, p.21) proposed what he described as "a pure number [...], freed of dependence upon any specific unit of measurement", the now-famous Cohen's d. Under the assumptions of normality and homogeneity of variance, Cohen's d (δ) is defined as the raw difference between two population means (μ_A and μ_B) divided by the common standard deviation (σ):

$$\delta = \frac{\mu_A - \mu_B}{\sigma} \tag{S1}$$

Cohen's d is a standardized measure of effect size that allows to express differences in terms of the variability of the phenomena of interest irrespective of the original measurement unit. It is a useful solution when researchers utilize raw units which are quite arbitrary or lack meaning outside their investigation (Cohen, 1988). A Cohen's d of 0.1 means that the difference between the two population means is one-tenth of the common standard deviation.

Borenstein et al. (2009) underline the importance of distinguishing between δ, the population Cohen's d value, and d, the estimated Cohen's d value from the sampled groups given by:

$$d = \frac{\bar{X}_A - \bar{X}_B}{S_{pooled}} \tag{S2}$$

In the numerator, $\bar{X}_A$ and $\bar{X}_B$ are the sample means in the two groups. In the denominator, S_{pooled} is the pooled standard deviation:

$$S_{pooled} = \sqrt{\frac{(n_A - 1)S_A^2 - (n_B - 1)S_B^2}{n_A + n_B - 2}} \tag{S3}$$

where n_A and n_B are the two sample sizes, and S_A^2 and S_B^2 are the standard deviations in the two groups.

Cohen's d interpretation

In well-established areas of study, the definition of a relevant effect size in terms of Cohen's d offers no particular difficulty. Population σ is normally already known or easy to estimate, and differences of interest are easily defined from the research context. Thus, researchers may already know which is the effect size of interest to evaluate, for example, the effectiveness of a specific treatment. On the contrary, in the case

of less known areas, or when newly developed measures are employed, the definition of an effect size of interest may not be so simple.

In these cases, Cohen (1988) proposed some conventional operational definitions to interpret effect sizes. He suggested indicative values of d for "small", "medium", and "large" effect sizes.

- *Small effect size: d = .2*; This refers to small differences that are difficult to detect, such as approximately the size of the difference in mean height between 15- and 16-year-old girls.
- *Medium effect size: d = .5*; This refers to differences that are "large enough to be visible to the naked eye" (p.26). For example, the magnitude of the difference in height between 14- and 18-year-old girls.
- *Large effect size: d = .8*; This refers to very obvious differences, such as the mean difference in height between 13- and 18- year-old girls.

Another way to interpret and make sense of Cohen's d values is to consider the Common Language effect size statistic (CL; Ruscio, 2008), or Cohen's measure of non-overlap U_3 (Cohen, 1988). The former is defined as the probability that a randomly chosen member of population B scores higher than a randomly chosen member of population A. The latter is defined as the percentage of the population B which exceeds the mean of the population A. Figure S1 shows CL and U_3 values for small, medium, and large effect sizes.

However, as suggested by Cohen (1988, p.25), "The terms *small*, *medium*, and *large* are relative, not only to each other but to the area of behavioural science or even more particularly to the specific content and research method being employed in any given investigation". These values are only conventional references in the absence of any other information. Researchers should aim to define their own criteria, according to their specific research objectives and the related costs-benefits ratio. In some fields, even small changes could result in valuable gains.

Finally, it is important to underline an aspect that is often neglected when dealing with Cohen's d. It should be remembered that Cohen's d depends on the pooled standard deviation (i.e., increasing levels of standard deviation are associated with lower values of Cohen's d). Given that the pooled standard deviation partly reflects the accuracy of the measure used in a study, in the planning phase researchers should select measures that are as accurate as possible. Furthermore, when evaluating effect sizes of other studies, considerations about the accuracy of the utilized measure(s) should always be taken into account.

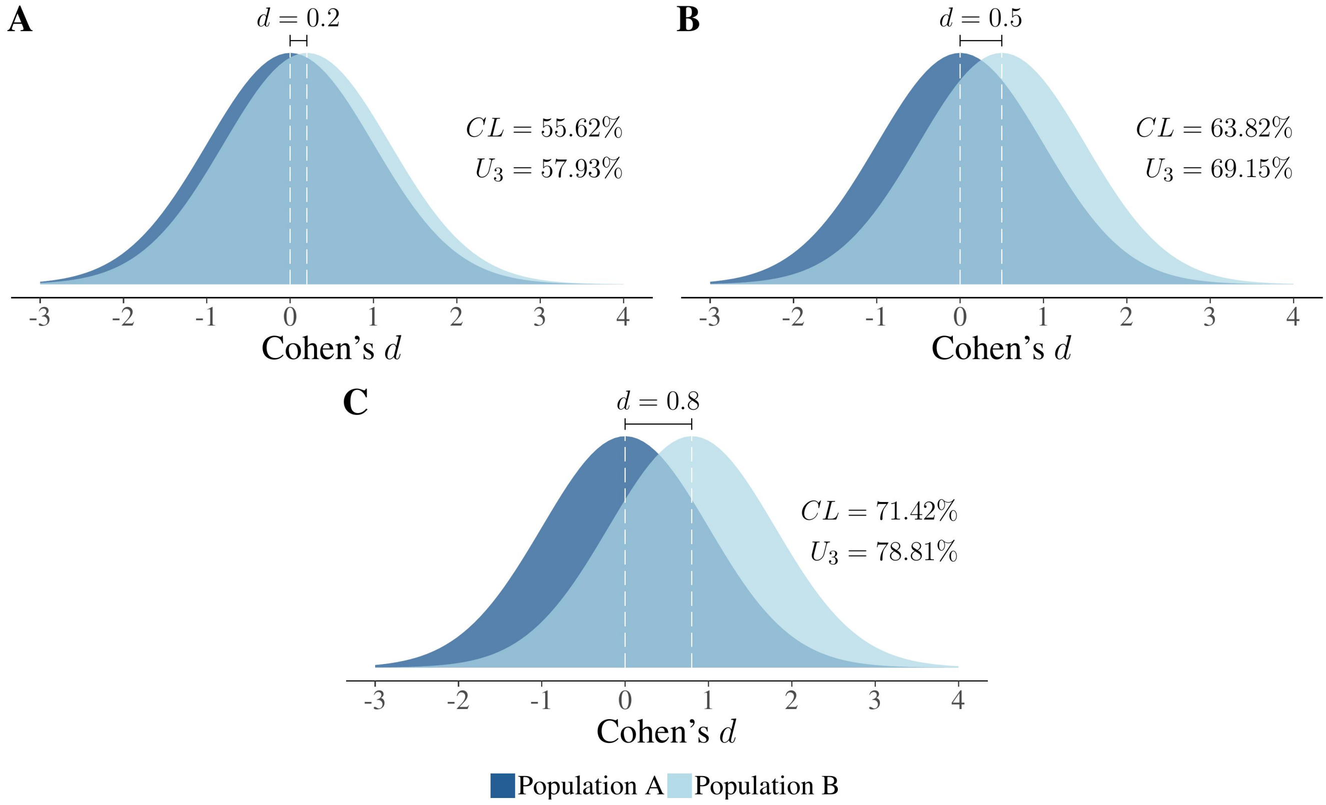

Figure S1. CL and U_3 values according to effect sizes. (**A**) In the case of a small effect ($d = .2$), there is a 56% probability that a random subject from population B has a higher score than a random subject from population A ($CL = .56$) and 58% of population B is above the mean of population A ($U_3 = .58$). (**B**) In the case of a medium effect ($d = .5$), there is a 64% probability that a random subject from the population B has a higher score than a random subject from population A ($CL = .64$) and 69% of population B is above the mean of the population A ($U_3 = .69$). (**C**) In case of a large effect ($d = .8$), there is a 71% probability that a random subject from population B has a higher score than a random subject from population A ($CL = .71$) and 79% of population B is above the mean of population A ($U_3 = .79$).

APPENDIX B: R FUNCTIONS FOR DESIGN ANALYSIS

Preliminary notes

The `R` functions presented in the paper to perform design analysis are:

- `design_analysis( )`
- `design_est( )`

These functions are detailed described in the following section, and their code (i.e., `PRDA.R`) is available at the Open Science Framework (OSF) at the link `https://osf.io/j8gsf/files/`.

In the last section of this Appendix, all examples in the paper are also reproduced using the aforementioned functions. It should be noted that results might slightly differ because both functions follow a simulation approach. To obtain more stable results, it is possible to increase the default number of iterations.

Readers can use the functions to easily perform prospective and retrospective design analysis on their own data[1].

Furthermore, the `R` code can be used as a starting point to extend design analysis to more complex cases than the one presented (i.e., the differences between two independent groups considering Cohen's d as an effect-size measure) and that were beyond the scope of this paper.

[1] Please note, that a first version of a package, called `PRDA`, to perform prospective and retrospective design analysis is also available at `https://github.com/masspastore/PRDA`.

R functions

To use our `R` functions, first download the file `PRDA.R` at the link `https://osf.io/j8gsf/files/`. To load the functions, simply type:

```
> source("PRDA.R")
```

Note: For the correct use of the functions, the `R` *package* `truncnorm` *(Mersmann et al., 2018) must be installed.*

design_analysis( )

<u>The function</u>

```
> design_analysis( d, n = NULL, power = NULL, sig.level = 0.05,
+                  rangen = c( 2, 1000 ), B = 1e4 , tol=.005 )
```

The function `design_analysis( )` runs prospective and retrospective design analysis according to a Cohen's d (`d`) and a fixed type I error (`sig.level`). Specifically, if the user specifies:

- power (`power`), then it performs *prospective design analysis*
- sample size per each group (`n`), then it performs *retrospective design analysis*

Note: It is necessary to provide either `power` *or* `n`*.*

<u>Function arguments</u>

- `d` = Cohen's d
- `n` = sample size for each group
- `power` = power
- `sig.level` = type I error. Default is .05
- `B` = number of replications for `d` simulation. Default is 10,000
- `rangen` = a vector of two values indicating the range of `n` within which to search for power. Default is from 2 to 1000.
  Note: `rangen` *is used only for prospective design analysis*
- `tol` = numerical tolerance used to search power. Default is .005.
  Note: `tol` *is used only for prospective design analysis*

<u>Function returned values</u>

- A list containing main arguments and results

Examples

```
> # Prospective design analysis
> design_analysis(d=.50, power=.80, sig.level=.05)
> # $d
> # [1] 0.5
> #
> # $power
> # [1] 0.8
> #
> # $n
> # [1] 64
> #
> # $typeS
> # [1] 0
> #
> # $typeM
> # [1] 1.132944
```

```
> # Retrospective design analysis
> design_analysis(d=.50, n=20, sig.level=.05)
> # $d
> # [1] 0.5
> #
> # $n
> # [1] 20
> #
> # $power
> # [1] 0.346
> #
> # $typeS
> # [1] 0.001156069
> #
> # $typeM
> # [1] 1.739772
```

design_est( )

The function

```
> design_est( n1 , n2 = n1, target_d = NULL, target_d_limits = NULL,
+             distribution = c("uniform","normal"), k = 1/6, sig.level = 0.05,
+             B = 500, B0 = 500, return_data = FALSE )
```

The function `design_est( )` performs retrospective design analysis according to a plausible interval for Cohen's d (see, `target_d_limits`) or to a fixed Cohen's d (see, `target_d`) and a fixed type I error (`sig.level`). Different sample sizes for each sample can be specified.
Note: It is necessary to provide either `target_d` *or* `target_d_limits`.

Function arguments

- `n1` = sample size of first group
- `n2` = sample size of second group. Default is `n1`
- `target_d` = Cohen's d.
- `target_d_limits` = vector of two values specifing the plausible interval of Cohen's d
- `distribution` = a character string specifying the probability distribution associated with the plausible interval for Cohen's d, must be one of `"uniform"` or `"normal"`
- `k` = if `"normal"` is specified as distribution, `k` is used to define the standard deviation of the doubly truncated normal distribution. Specifically, the standard deviation is calculated as the length of the plausible interval times `k`. Default is $\frac{1}{6}$
- `sig.level` = type I error. Default is .05
- `B` = number of replications for Cohen's d simulation. Default is 500
- `B0` = number of Cohen's d sampled from the plausible interval. Default is 500
- `return_data` = if `TRUE` and a plausible interval for Cohen's d is specified, it returns a `data.frame` of `B0` rows with power, Type S and Type M errors for each Cohen's d sampled from the plausible interval. Default is `FALSE`

Function returned values Two lists:

- `class` containing the main arguments
- `results` containing the results

Examples

```
> # Retrospective design analysis with plausible interval
> #  and associated doubly truncated normal distribution
> out <- design_est(n1=50, n2=48, distribution="normal",
+                  target_d_limits = c(.20,.30), return_data=TRUE)
> #
> out
> # $call
> # $call$n1
> # [1] 50
> #
> # $call$n2
> # [1] 48
> #
> # $call$target_d_limits
> # [1] 0.2 0.3
> #
> # $call$distribution
> # [1] "normal"
> #
> # $call$k
> # [1] 0.1666667
> #
> # $call$B
> # [1] 500
> #
> # $call$B0
> # [1] 500
> #
> #
> # $results
> # $results$power
> # [1] 0.232588
> #
> # $results$typeS
> # [1] 0.003919314
> #
> # $results$typeM
> # [1] 2.090674
> #
> # $results$data
> #     power       typeS    typeM
> # 1   0.240 0.000000000 2.024220
> # 2   0.162 0.000000000 2.045419
> # 3   0.236 0.000000000 2.104194
> # 4   0.188 0.010638298 2.048976
> # 5   0.208 0.000000000 2.062765
> # ...
> #
> #
> # To draw the distribution of simulated power, Type S and Type M errors:
> hist(out$results$data$power,main="",xlab="power") # power
> hist(out$results$data$typeS,main="",xlab="typeS") # Type S error
> hist(out$results$data$typeM,main="",xlab="typeM") # Type M error
```

Examples included in the paper

```
> # Clear workspace
> rm(list=ls())
> # Load the functions for design analysis
> source("PRDA.R")
```

```
> # Subsection 1.3
> design_analysis(n=33, d=.20)
```

```
> # Subsection 2.1
> design_analysis(d=.25, power=.80)
> design_analysis(d=.25, power=.60)
```

```
> # Subsection 2.2
> design_analysis(n=31, d=.25)
```

```
> # Section 3
> design_est(n1=31, n2=31, target_d_limits=c(.20,.60), distribution="normal")
```

```
> # Section 4: Prospective design analysis
> power1=.80 ; power2=.60
> d1=.20 ; d2=.35 ; d3=.50
> design_analysis(d=d1, power=power1)
> design_analysis(d=d2, power=power1)
> design_analysis(d=d3, power=power1)
> design_analysis(d=d1, power=power2)
> design_analysis(d=d2, power=power2)
> design_analysis(d=d3, power=power2)
> # Section 4: Retrospective design analysis
> design_est(n1=34,n2=33,target_d=d1,B=10000)
> design_est(n1=34,n2=33,target_d=d2,B=10000)
> design_est(n1=34,n2=33,target_d=d3,B=10000)
> # Section 4: Retrospective design analysis with plausible interval
> design_est(n1=34, n2=33, distribution="normal",target_d_limits = c(.20,.50))
```

For any further information please contact:
`massimiliano.pastore@unipd`, `gianmarco.altoe@unipd.it`

REFERENCES

Borenstein, M., Hedges, L. V., Higgins, J. P. T., and Rothstein, H. R. (2009). *Introduction to meta-analysis* (Chichester, U.K: John Wiley & Sons). doi:10.1002/9780470743386

Cohen, J. (1988). *Statistical Power Analysis for the Behavioral Sciences* (New York, NY: Lawrence Erlbaum Associates). doi:10.4324/9780203771587

Mersmann, O., Trautmann, H., Steuer, D., and Bornkamp, B. (2018). *truncnorm: Truncated Normal Distribution*. R package version 1.0-8. Available at `https://CRAN.R-project.org/package=truncnorm`

Ruscio, J. (2008). A probability-based measure of effect size: Robustness to base rates and other factors. *Psychological Methods* 13. doi:10.1037/1082-989X.13.1.19
